# Epidemiology of Ticks and Tick‐Borne Hemopathogens of Cattle in Two Selected Districts of Northwest Ethiopia

**DOI:** 10.1002/vms3.70293

**Published:** 2025-03-03

**Authors:** Aschalew Shitu Yenew, Shimelis Dagnachew Nigatu, Zewdu Seyoum, Mersha Chanie

**Affiliations:** ^1^ Department of Veterinary Biomedical and Paraclinical Sciences, School of Veterinary Medicine Bahir Dar University Bahir Dar Ethiopia; ^2^ College of Veterinary Medicine and Animal Sciences University of Gondar Gondar Ethiopia

**Keywords:** cattle, haemopathogens, Northwest Ethiopia, season, tick

## Abstract

**Background:**

Ticks and tick‐borne haemopathogens are major obstacles to cattle production causing significant economic losses in Ethiopia.

**Objective:**

The objective of this study was to determine the epidemiology of ticks and tick‐borne haemopathogens in cattle in Northwest Ethiopia.

**Methods:**

Cross‐sectional studies were conducted in dry and short rainy seasons. A stratified random sampling technique was employed. Accordingly, a total of 392 cattle were examined. During sampling parameters like; sex, age, breed, body condition score, district and production system were recorded for each animal. Ticks were collected from each animal and examined under stereomicroscope for species identification. The blood sample was taken from the ear vein by pricking with a lancet and then thin smear was made with a frosted microscopic slide and stained with Giemsa for the detection of haemopathogen infections with 100× oil immersion magnification.

**Results:**

Of the 392 cattle examined, 87.8% and 17.1% were positive for tick infestation and tick‐borne haemopathogens, respectively. The prevalence of tick and tick‐borne haemopathogens was 93.9% and 27% in the short rainy season and 81.6% and 7.1% in the dry season, respectively. *Amblyomma*, *Rhipicephalus*, *Hyalomma* and *Rhipicephalus* (*Boophilus)* genera, and *A. variegatum*, *A. lepidum*, *Rh. evertsi*, *Hy. rufipes* and *Rh. (B.) decoloratus* species were identified. *Rhipicephalus (B.) decoloratus* was the most prevalent (66.1%) and abundant (38%) tick species. Season, district, age and sex showed significant (*p* < 0.05) associations with tick infestations.

*Babesia bigemina* (10%), *Anaplasma marginale* (5.4%), *Theileria* species (3.1%) and *Anaplasma centrale* (1.3%) haemopopathogens were detected. Except, for *Anaplasma central*, they had a significant (*p* < 0.05) association with season.

**Conclusion:**

Ticks and tick‐borne haemopathogens were found to be prevalent and had seasonal dynamics. Therefore, strategic and integrated control approaches against the vector and the parasite should be designed.

## Graphical abstract

- The overall prevalence of tick infestation and tick‐borne haemopathogens of cattle in the study area was 87.8% and 17.1%, respectively.
- Five species of ticks under three genera were detected; namely: *Amblyomma variegatum, Amblyomma lepidum, Rhipicephalus evertsi, Hyalomma rufipes* and *Rhipicephalus decoloratus* were identified.
- *Babesia bigemina*, *Anaplasma marginale*, *Anaplasma centrale* and *Theileria* species of haemopopathogens were also detected in the area.

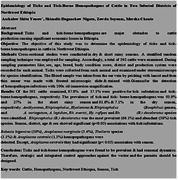

## 1 Introduction

Livestock, especially cattle, play a paramount role in agricultural production systems, particularly in poor countries throughout the world (De Meneghi et al. 2016). Ethiopia has a large livestock population with an estimated 61.51 million cattle, 33.02 million sheep, 38.96 million goats, 1.76 million camels, 11.96 million equines and 59.42 million poultry, which represent immense economic potential (CSA 2019). However, the production and contribution of this massive livestock resource to the country's national income are unduly small due to several factors, such as parasites (Ashenafi et al. 2013). Among these ticks are the most important external parasites of cattle (Nyangiwe et al. 2018). In Ethiopia, ticks are directly or indirectly involved in causing considerable financial losses to the livestock industry accounting for 75% of the animal exports (Zeleke and Bekele 2004), and about 53,419USD losses annually through rejection and down‐grading of hides and skins in the country (Ashenafi et al. 2013). Ticks are common in all agroecological zones of the country. In Ethiopia, ticks occupy the first rank among external parasites and the economic losses incurred when they infest livestock, particularly cattle. In contrast to this huge economic loss caused by ticks, some of the owners neglect ticks as an animal health problem, most of them have little knowledge about the effect of ticks on their animals and few know about diseases transmitted to domestic animals by ticks (Abunna et al. 2009). The main tick genera reported in Ethiopia are *Amblyomma*, *Rhipicephalus*, *Hyalomma* and *Haemaphysalis* with more than 60 species of ticks infesting both domestic and wild animals. Among these, about 37 species are widespread and transmit important parasites of livestock (Tomassone et al. 2012). Previous studies on the prevalence of tick infestation on cattle in different parts of Ethiopia reported 82% in the Bedele district of western Ethiopia (Beyene et al. 2015); 61% in the Humbo district of southern nations and nationalities, Ethiopia (Wasihun and Doda 2013); 96.4% in and around Chiro town, Oromia region (Seid and Mohammed 2017) and 63% in Borecha district, Southern Ethiopia (Kemal et al. 2017).

Ticks are known to transmit more pathogens than any other group of arthropods worldwide. They transmit disease‐causing agents for babesiosis, anaplasmosis, theileriosis and cowdriosis (Nyangiwe et al. 2018). For instance, *Rhipicephalus (Boophilus) decoloratus* transmits *Babesia bigemina* (Horak et al. 2017), *Anaplasma marginale* and *Borrelia theileri* in cattle (Madder et al. 2013). Similarly, *Rhipicephalus evertsi* transmits *Anaplasma marginale* in cattle (Horak et al. 2017; Bock et al. 2004). It also transmits the cause of spirochetosis (*Borrelia theileri*) and *Babesia bigemina* in cattle (Madder et al. 2013). *Amblyomma variegatum*, the tropical bont tick, transmits the agents of heartwater (*Ehrlichia ruminantium)*, benign bovine theilerioses (*Theileria mutans*, *Theileria velifera*), bovine ehrlichiosis (*Ehrlichia bovis*) (Madder et al. 2013). *Anaplasma marginale* also is transmitted by *Hyalomma rufipes* in cattle (Tadesse et al. 2012).

About 80% of the world's cattle population is at risk of ticks and tick‐borne diseases (TTBDs), causing a global annual loss of $US 22–30 billion (Nyangiwe et al. 2018). Tick‐borne diseases represent an important proportion of all animal diseases affecting the livelihood of poor farmers in tropical countries (FAO 2011). Due to the economic and veterinary importance of ticks, the control and the transmission of tick‐borne diseases (TBDs) remain a challenge for the cattle industry in tropical and subtropical areas of the world (Lodos et al. 2000). The use of acaricides for the treatment of ectoparasites has become a serious global problem due to the development of resistance and environmental pollution (Ahmed et al. 2023). However, in Ethiopia, acaricides are still used widely for the control of ticks as well as TBDs (Abdela 2016). Even though there are a number of options to control TBDs, the main one is controlling ticks (vectors) by using chemical acaricides. In Ethiopia, TBDs contribute the most to animal diseases. It is an obstacle to the introduction of highly productive exotic breeds into different regions of the country (Abunna et al. 2009). Tick‐borne haemopathogens are a very high burden on cattle in Ethiopia. For instance, 96.9% of prevalence was reported in the southwestern part (Hailemariam et al. 2017) and 3.9% in and around Debre Zeit, Central Ethiopia (Sitotaw et al. 2014). Even though numerous studies are performed on the identification and prevalence of TTBDs in many parts of Ethiopia there is a shortage of recent information on species and seasonal dynamics of TTBDs in the present study areas. Therefore, the aim of the present study was to assess the epidemiology of tick infestations and tick‐borne haemopathogens of cattle in the Bahir Dar Zuria and Guangua districts of Northwest Ethiopia.

## 2 Methods

### 2.1 Description of Study Area

The study was conducted in the Bahir Dar Zuria and Guangua districts, including the two ranches (Andassa Livestock Research Center (ALRC) and Chagni Cattle Breeding and Improvement Ranch, respectively.

Bahir Dar Zuria district is located at a distance of 564 km northwest of the capital city Addis Ababa. It is situated at an altitude ranging from 1700 to 2300 m above sea level (masl), and it receives an average annual rainfall (RF) ranging from approximately 820 to 1250 mm. The minimum and maximum daily temperatures of the area are 10°C and 32°C, respectively. It has 86,976 cattle, 37,645 sheep, 22,853 goats, 13,848 equines and 687,593 poultry population (BZDoA, 2018). The ALRC, a previous Andassa cattle breeding and multiplication ranch, was established in 1964 by the USA point‐four project to conserve and improve Fogera cattle. It is situated in the heart of the Blue Nile, 22 km away from the capital city of the Amhara region, Bahir Dar on the road to Tis Abay. It is located at 11°29'N latitude and 37°29'E longitude with an elevation of 1730 masl. It receives an average annual RF of 1150 mm with temperatures ranging from 6.5°C to 30°C. The centre lies on 360 hectares of land, of which 310 hectares are used for grazing land, hay production and irrigation activities (ALRC 2017).

**FIGURE 1:**
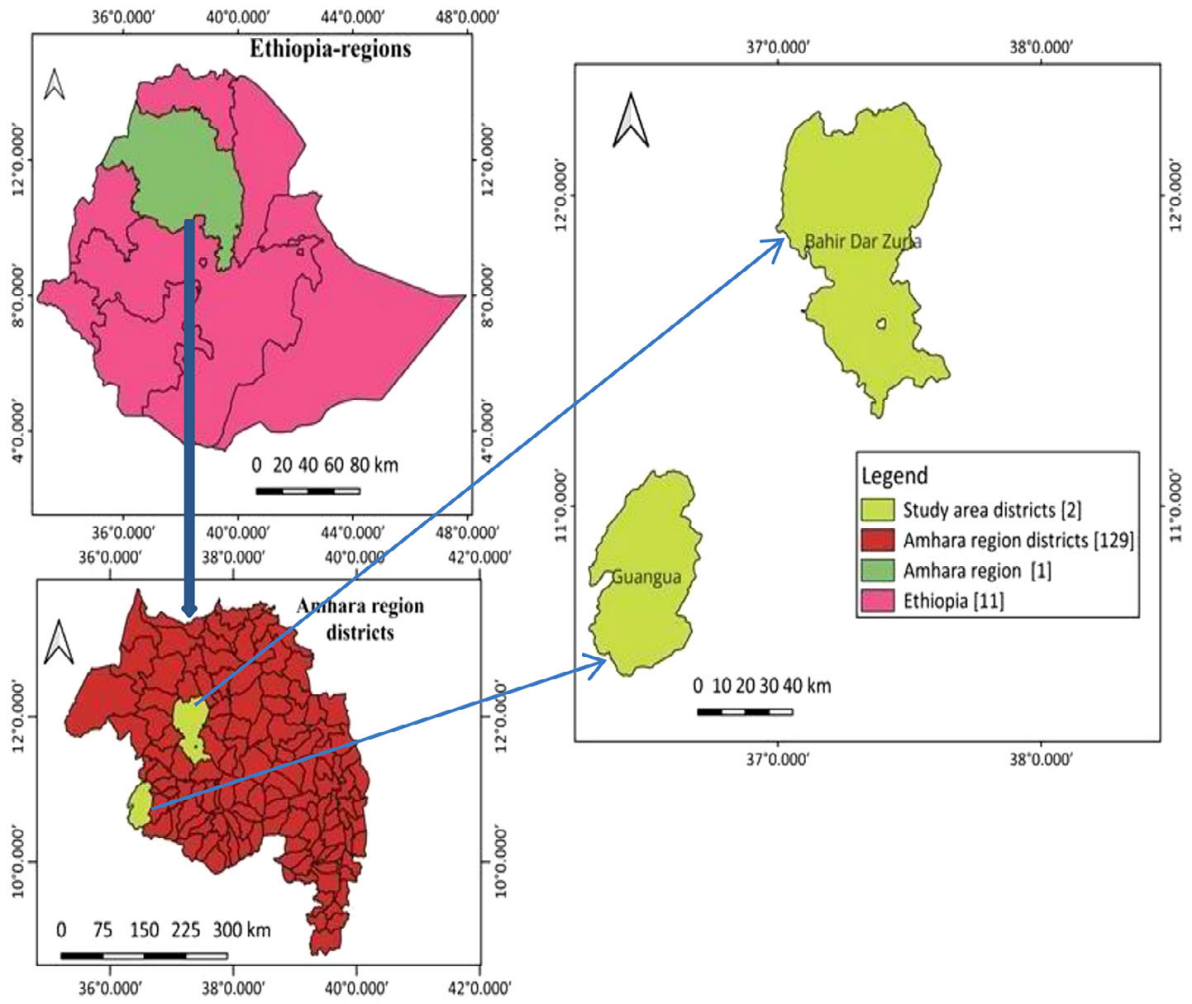
Study area location map.

Guangua district is located at a latitude of 11°00′ N and longitude of 36°20′00ʺ E, which is part of the Awi zone, Amhara region, Ethiopia. It is bordered on the south and west by the Benishangul‐Gumuz region. The Guangua district has an area of 106,914 hectares of land surface, which consists of 60% plain, 28% hilly and the remaining 12% gorges and valleys. It has an altitude ranging from 1500 to 2000 masl. The area gains a mean annual RF of 1365 mm, and the minimum and maximum annual temperatures are 22°C and 27°C, respectively. It has 145,851 cattle, 35,740 sheep, 22,955 goats, 12,948 equines and 287,593 poultry populations. Chagni ranch is found near Chagni town, which has a latitude and longitude of 10°57′N and 36°30′E, respectively, and an elevation of 1583 masl (GDoA, 2014) (Figure 1).

### 2.2 Study Population

The study population was all cattle irrespective of their sex, age, breed, body condition score (BCS) and production system in the study areas. The sampled animals were categorized according to their body condition according to Nicholson and Butterworth (1986) as poor (BCS 1 and 2), medium (BCS 3) and good (BCS 4 and 5). Cattle that are extremely lean, having prominent dorsal spines pointed to the touch and individual visible transverse processes into which a finger could be easily pushed, were categorized as having a poor BCS. Medium BCS cattle have visible ribs with little fat cover and barely visible dorsal spines. Animals that had fat cover easily visible in critical areas and transverse processes that were not seen or felt were categorized as having a good BCS. Categorization of cattle was also performed on age (< 2 years = young and ≥ 2 years = adult), sex (male and female), breed (local and cross) and management system (extensive, semi‐intensive and ranch).

### 2.3 Study Design

A cross‐sectional study was conducted twice during the study period (December 2018 to June 2019) in selected districts of western Amhara (Bahir Dar Zuria and Guangua districts) to assess the seasonal (dry‐January to February and short rainy season‐April to June) dynamics of ticks and tick‐borne haemopathogens. The two districts were selected purposively to include the two ranches (Andassa and Chagni), which are found in the study districts.

### 2.4 Sampling Methods and Sample Size Determination

The samples were selected using a stratified random sampling technique. Seven peasant associations were selected randomly from the Bahir Dar Zuria and Guangua districts. The production system was stratified into ranches, extensive and semi‐intensive. Animals were also selected randomly from different strata with a proportional allocation method from selected farmer associations and ranches. The sample size needed for this study was determined with the expected prevalence of TBDs at 15%, taking the average prevalence reported by Hamsho et al. (2015) and Choramo and Ibrahim (2017). The sample size was calculated using the formula forwarded by Thrusfield (2012) using a 95% confidence interval and 5% absolute precision. A total of 392 cattle and 196 animals during each season were examined.

$$N=\frac{Z^{2}P_{\exp}\left(1-P_{\exp}\right)}{d^{2}}$$

where, *N* is needed sample size, *Z*α/2 is the *Z* (normal distribution) value for a chosen confidence level, *P*_exp_ is expected prevalence, and *d* is desired absolute precision

$$N=\frac{1.96^{2}0.15\left(1\;-\;0.15\right)}{0.05^{2}}$$

Therefore, the sample size for this study was 196 animals per season.

### 2.5 Sample Collection and Identification

#### 2.5.1 Tick Collection and Identification

Each sampled animal was well restrained before examination and collection of ticks. Then, the entire parts of each animal were inspected for tick infestation. All detected ticks were removed from the host by using strong steel forceps, which have blunt ends and serrated inner surfaces. The ticks were gripped firmly over the scutum and mouth parts and then pulled directly out to prevent damage to the mouthparts. The ticks removed from different infestation sites (dewlap, groin and udder/scrotum, brisket and axillae, under tail and anal area, neck and head area, belly and legs, and lateral and dorsal part) of the host were collected in separate glass bottles containing 70% ethanol and labelled with the date of collection, species, sex, breed, age of the host, place of collection and tick infestation site as stated by (Latif and Walker 2016), and body condition of the animal and production system were included. The collected ticks were transported to the Bahir Dar Animal Health Diagnostic and Investigation Laboratory (BAHDIL) for identification at the species level under a stereomicroscope based on their size, mouthparts, the colour of legs or scutum/conscutum, festoon enamelling, presence or absence of eye and other features of tick species (Walker et al. 2014).

#### 2.5.2 Blood Sample Collection and Examination of Tick‐Borne Haemopathogens

Blood samples were collected from the ear vein after disinfecting the area with 70% ethyl alcohol. When the area was dried, the ear vein was pricked with a lancet, the first drop of blood was taken with a frosted clean microscopic slide, and then a thin smear was made immediately. Each smear was labelled with a code that represents the date of collection, species, sex, breed, age, body condition, production system of the host and place of collection. After labelling, the prepared slides were fixed with absolute methanol and transported to BAHDIL. Finally, the slide smear was stained with 10% Giemsa for examination of tick‐borne haemopathogens under an oil immersion lens (100×).

### 2.6 Statistical Analyses

The data were coded and inserted into a Microsoft Office Excel spreadsheet and checked before analysis using STATA Corp LP software version 14. Descriptive statistics were used to summarize data in the form of graphs and tables. The associations of tick infestation and TBDs with respect to their risk factors were analysed by unavailable and multivariable logistic regression. Tick infestation burden was analysed with negative binomial regression. *p* values less than 0.05 were considered statistically significant.

## 3 Results

### 3.1 Tick Species and Prevalence

**FIGURE 2:**
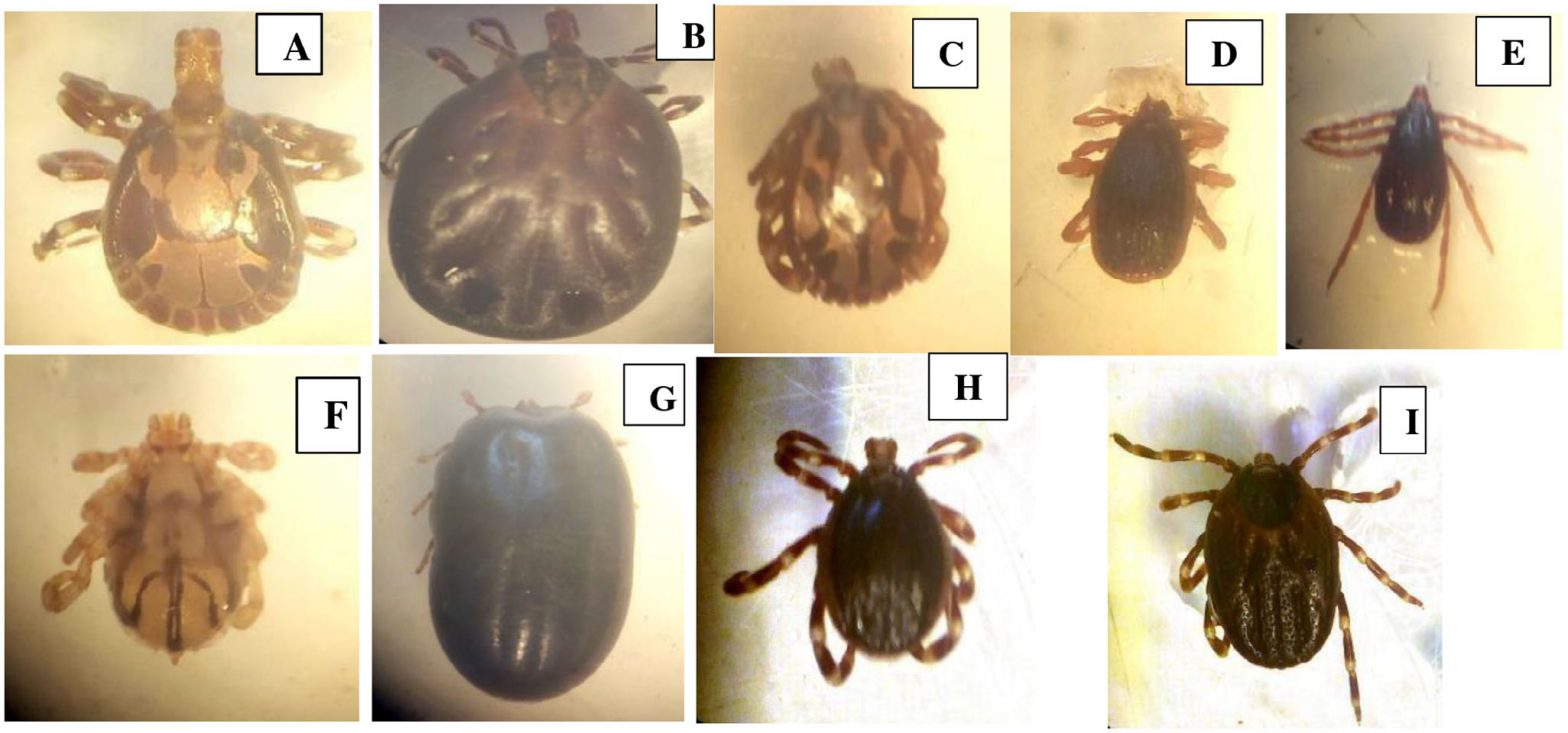
Photographs of hard ticks collected from Bahir Dar Zuria and Guangua districts of Northwest Ethiopia during the study period: *A. variegatum*, male (A) *A. variegatum*, female (B) *A. lepidium*, male (C) *R. evertsi*, male (D) *R. evertsi*, female (E) *R. (B.) decoloratus*, male (F) *R. (B.) decoloratus* female (G) *Hy. rufipes*, male (H) *Hy. rufipes*, female (I).

Of the 392 cattle examined for tick infestation, 87.8% (344/392) were infested with one or more type(s) of tick species. Five tick species under four genera were identified in the study area, as shown below in Figure 2. *Rhipicephalus (Boophilus), Amblyomma, Rhipicephalus* and *Hyalomma* genera were identified. Under these genera, *Rh. (B.) decoloratus*, *A. variegatum*, *A. lepidum*, *Rh. evertsi* and *Hy. rufipes* were also recorded.*Rhipicephalus (B.) decoloratus*was the first most prevalent tick species, with a prevalence of 66.1%, followed by *Rh. evertsi*at 51.0*%, A. variegatum* at 50.3%, *Amblyomma nymph*14.8%,*Hy. rufipes* 14.5%, *Rh*. *(Boophilus) nymph* 10.7% and *A. lepidum* 1.0% in descending order. During the entire period of study, 4329 adult and 710 immature ticks were collected.

### 3.2 Tick Abundance and Infestation Burden

**FIGURE 3:**
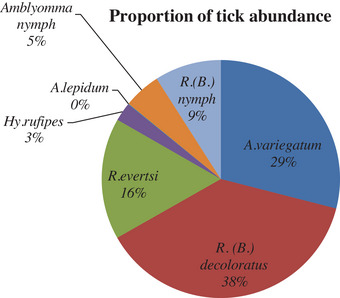
Overall relative abundance of ticks.

During the entire period of study, 4329 adult and 710 immature ticks were collected. Among these 1904 (38%), 1461 (29%), 832 (16%), 456 (9%), 254 (5%), 128 (3%) and 4 (0%) were *Rh. (B.) decoloratus*, *A. variegatum*, *Rh. evertsi*, *Rh. (Boophilus) nymph*, *Amblyomma nymph*, *Hy. rufipes* and *A. lepidum*, respectively. *Rh. (B.)* *decoloratus*species was the most dominant tick species, whereas *A. lepidum* was the least abundant tick species in the study areas (Figure 3). The tick infestation burden was different in different categories of each factor (Table 1). Only season and district had statistically significant (*p* < 0.001) differences in tick infestation burden. Tick infestation burden was expressed in terms of total mean tick count per animal. The tick count per animal was significantly higher (*p* < 0.001) during the short rainy season (19.12 ± 2.82) than during the dry season (4.60 ± 0.70). Similarly, the mean tick count per animal was significantly (*p* < 0.001) higher in Guangua district (12.10 ± 1.86) than in Bahir Dar Zuria district (7.26 ± 1.08). However, tick infestation burden was not significant (*p* > 0.05) among production system, BCS, breed, age and sex. The tick count per animal was not significantly different (12.81 ± 2.14, 10.32 ± 1.40 and 6.23 ± 2.15) in the extensive, ranch and semi‐intensive production systems, respectively.

**TABLE 1:** Mean tick infestation burden (mean ± SE) analyzed by negative binomial regression.

Factors	Category	Mean ± SE	95% CI		*p* value
			Lower	Upper	
Season	Dry	4.60 ± 0.70	3.23	5.96	0.000^b^
	Short rainy^a^	19.12 ± 2.82	13.59	24.64	
District	Bahir Dar Zuria	7.26 ± 1.08	5.14	9.38	0.000^b^
	Guangua^a^	12.10 ± 1.86	8.45	15.75	
Production system	Extensive	12.81 ± 2.14	8.63	17.00	0.072
	Ranch	10.32 ± 1.40	7.57	13.07	0.177
	Semi‐intensive^a^	6.23 ± 2.15	2.02	10.44	
BCS	Good	11.20 ± 2.72	5.88	16.53	0.126
	Medium	9.63 ± 1.23	7.21	12.05	0.102
	Poor^a^	7.63 ± 1.25	5.18	10.09	
Breed	Cross	11.234 ± 2.25	6.83	15.63	0.122
	Local^a^	7.82 ± 1.25	5.37	10.27	
Age	Young	10.09 ± 1.73	6.70	13.48	0.352
	Adult^a^	8.71 ± 1.27	6.22	11.20	
Sex	Male	8.98 ± 1.41	6.20	11.75	0.482
	Female^a^	9.79 ± 1.41	7.02	12.56	

Abbreviations: CI = confidence interval, SE = standard error.

^a^ reference category.

^b^ statistically significant.

### 3.3 Seasonal Dynamics of Ticks

**FIGURE 4:**
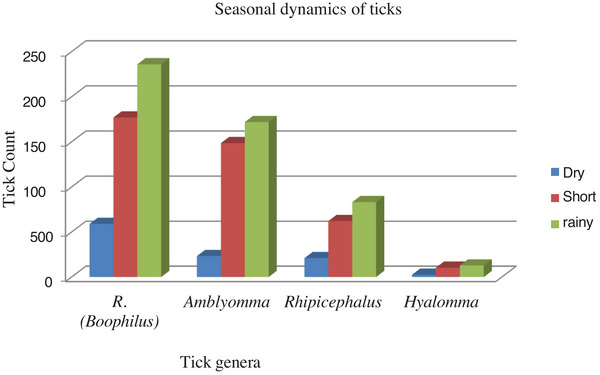
Bar graph showing seasonal dynamics of tick genera.

All tick genera increased during the short rainy season as shown below in Figure 4. Generally, almost all ticks except *Amblyomma* nymphs increased in abundance in the short rainy season. Furthermore, *A. variegatum*, *Rh. evertsi*, *Hy. rufipes* and *Rh. (B.)* nymphs increased significantly in the short rainy season. *A. variegatum*, *Rh. evertsi*, *Hy. rufipes* and *Rh. (B.)* nymph infestations were 10.3 (*p* < 0.001), 1.9 (*p* < 0.05), 2.98 (*p* < 0.05) and 11.5 (*p* < 0.001) times more likely to occur in the short rainy season than in the dry season (Table 2).

**TABLE 2:** Seasonal dynamics of tick species/genus.

Tick species/genus	Season	OR	SE	Overall prevalence (%)	*p* value	95% CI
*R. (B.)decoloratus*	Dry^a^					
	Short rainy	0.98	0.21	66.1	0.915	0.64–1.48
*A. variegatum*	Dry^a^					
	Short rainy	10.34	2.45	50.3	0.000^b^	6.49–16.46
*R. evertsi*	Dry^a^					
	Short rainy	1.93	0.40	51	0.001^b^	1.29–2.89
*Hy. rufipes*	Dry^a^					
	Short rainy	2.98	0.94	14.5	0.001^b^	1.61–5.51
*A. lepidum*	Dry^a^					
	Short rainy	3.03	3.51	1	0.339	0.31–29.40
*Amblyomma nymph*	Dry^a^					
	Short rainy	0.78	0.22	14.8	0.394	0.45–1.37
*R. (Boophilus) nymph*	Dry^a^					
	Short rainy	11.54	6.19	10.7	0.000^b^	4.03–33.04

Abbreviations: CI = confidence interval, OR = odds ratio, SE = standard error.

^a^ reference category.

^b^ statistically significant.

### 3.4 Risk Factors Associated With Tick Infestation

Risk factors considered, including season, district, production systems, BCS, breed, sex and age, were analysed in univariable logistic regression. Accordingly, season, district, breed, age and sex were significantly (*p* < 0.05) associated with tick infestation (Table 3). These significant risk factors were further analysed with multivariable logistic regression (Table 4). Consequently, district, season, age and sex of the animal had a significant association with tick infestation. Cattle in Guangua district were 48.65 times more likely to be at risk of tick infestations than those in Bahir Dar Zuria district. Likewise, tick infestations were 4.90 times more likely to occur higher in the short rainy season than in the dry season. Adult cattle were 3.43 times more likely to be at risk of tick infestation than young cattle, and female animals were also 2.6 times more likely to be at risk than male animals (Table 4).

**TABLE 3:** Univariable logistic regression analysis of risk factors associated with tick infestations.

Risk factors	Category	No. of examined animals	No. of infested animals (Prevalence %)	COR (95% CI)	*p* value
Season	Dry^a^	196	160 (81.6)		
	Short rainy	196	184 (93.9)	3.45 (1.74–6.86)	0.000^b^
District	Bahir	145	100 (67)		
	Dar Zuria^a^				
	Guangua	247	244 (98.8)	36.6 (11.12–120.5)	0.000^b^
Production	Extensive^a^	213	191 (89.7)		
system	Ranch	167	145 (86.8)	0.76 (0.4–1.42)	0.391
	Semi‐intensive	12	8 (66.7)	0.23 (0.06–0.83)	0.024^b^
	Good^a^	24	20 (83.3)		
BCS	Medium	279	241 (86.4)	1.27 (0.41–3.91)	0.679
	Poor	89	83 (93.3)	2.77 (0.71–10.74)	0.141
	Cross^a^	40	28 (70)		
Breed	Local	352	316 (89.8)	3.76 (1.76–8.04)	0.001^b^
	Young^a^	109	86 (78.9)		
Age	Adult	283	258 (91.2)	2.76 (1.49–5.11)	0.001^b^
	Male^a^	140	114 (81.4)		
Sex	Female	252	230 (91.3)	2.38 (1.29–4.39)	0.005^b^

Abbreviations: CI = confidence interval, COR = crude odds ratio.

^a^ reference category.

^b^ statistically significant.

**TABLE 4:** Multivariable logistic regression analysis of risk factors associated with tick infestations.

Risk factors	Category	No. of examined animals	No. of infested animals (prevalence %)	AOR (95% CI)	*p* value
Season	Dry^a^	196	160 (81.6)		
	Short rainy	196	184 (93.9)	4.9 (2.19–10.94)	0.000^b^
District	Bahir Dar	145	100 (69)		
	Zuria^a^				
	Guangua	247	244 (98.8)	48.65 (13.79–171.7)	0.000^b^
Age	Young^a^	109	86 (78.9)		
	Adult	283	258 (91.2)	3.43 (1.51–7.76)	0.003^b^
Sex	Male^a^	140	114 (81.4)		
	Female	252	230 (91.3)	2.26 (1.03–4.96)	0.041^b^

Abbreviations: AOR = adjusted odds ratio, CI = confidence interval.

^a^ reference category.

^b^ statistically significant.

### 3.5 Infestation Site Preference

**FIGURE 5:**
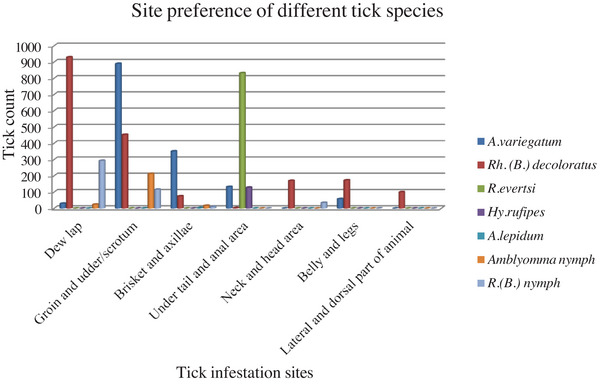
Infestation sites of different tick species.

Different tick species have different preferences for infesting specific body parts of the host. Even though *Rh. (B.) decoloratus* and *A. variegatum* were collected from different parts of the host, and they mainly dominated dewlap, groin and udder/scrotum, respectively, *Rh. evertsi* and *Hy. rufipes* were found only under the tail and anal areas (Figure 5).

### 3.6 Male/Female Sex Ratio

From 4329 adult ticks collected, 2269 (52.4%) were males and 2060 (47.6%) were females. Apart from *Rh. (B.) decoloratus*, the remaining tick species were mainly dominated by males (Table 5).

**TABLE 5:** Male/female sex ratio of different tick species.

	Tick count			
Tick species	Male	Female	Total	Sex ratio (M/F)
*A. variegatum*	1288	173	1461	7.45:1
*Rh.(B.) decoloratus*	331	1573	1904	0.21:1
*R. evertsi*	552	280	832	1.97:1
*Hy. rufipes*	94	34	128	2.76:1
*A. lepidum*	4	0	4	4:0
Overall	2269	2060	4329	1.1:1

### 3.7 Tick‐Borne Haemopathogen Species and Prevalence

**FIGURE 6:**
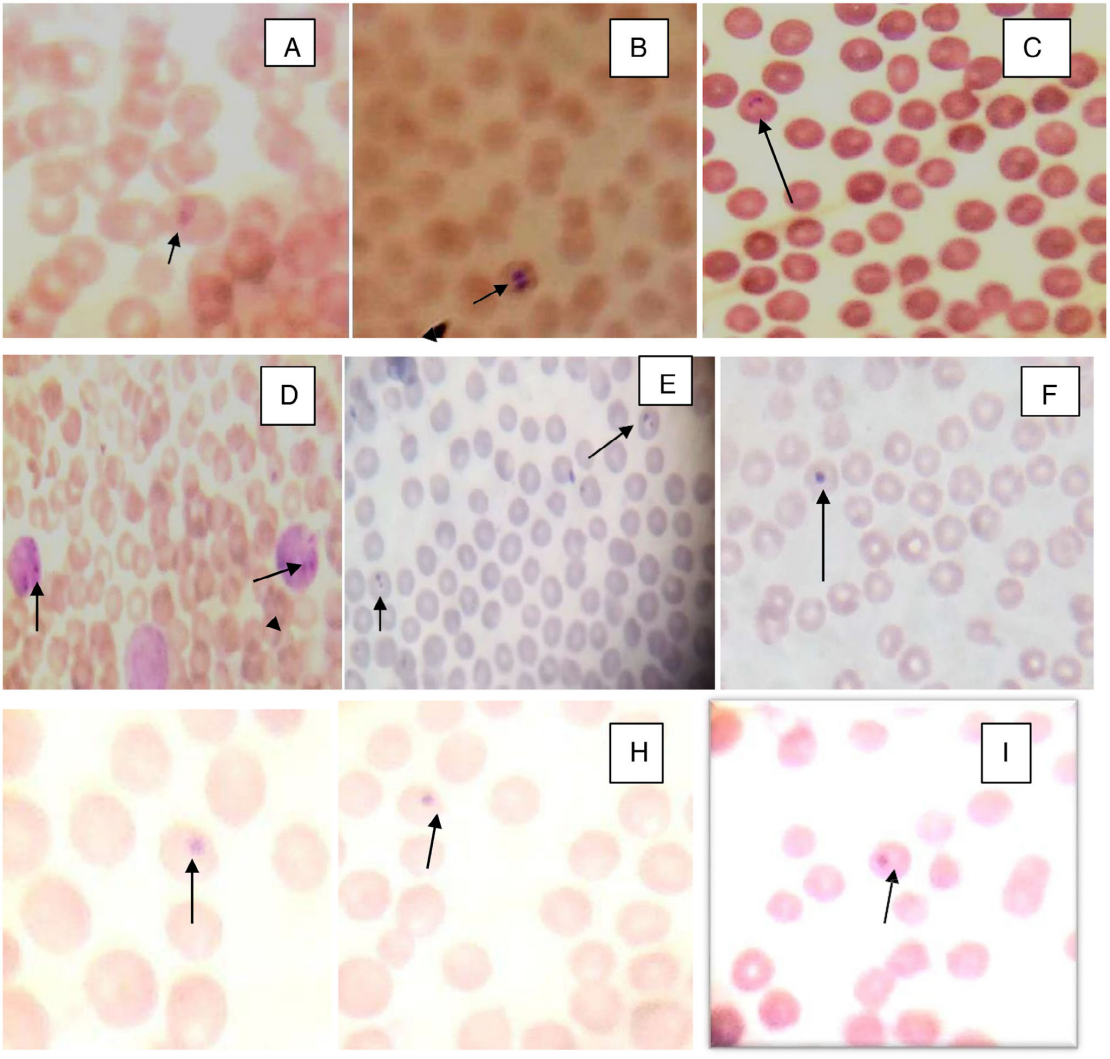
Photo‐micrograph of tick‐borne haemopathogens (*Babesia bigemina* (A, B, C), *Theileria* species within lymphocytes (D), *Theileria* species within erythrocytes (E), *Anaplasma centrale* (F), *Anaplasma marginale* (G, H, I)) during the study period from Bahir Dar Zuria and Guangua districts of Northwest Ethiopia.

Out of 392 cattle examined for tick‐borne haemoparasites, 17.1% (67/392) were infected with one or more tick‐borne haemopathogens. *Babesia bigemina*, *A. marginale*, *A. centrale* and *Theileria*sp. were detected from Giemsa‐stained thin blood smears (Figure 6). *Babesia bigemina* was the most prevalent tick‐borne haemopathogen, followed by *A. marginale, Theileria*sp. and *A. centrale* in descending order.

#### 3.7.1 Seasonal Dynamics of Tick‐Borne Haemopathogens

All tick‐borne haemopathogens were increased in the short rainy season compared with the dry season, but *B. bigemina*, *A. marginale* and *Theileria*sp. only were statistically significant (*p *< 0.05). *Babesia bigemina* was 3.23 times more likely to occur in the short rainy season than in the dry season. Similarly, *A. marginale* and *Theileria*sp. were also 6.51 and 5.22 times more likely to occur during the short rainy season than during the dry season, respectively (Table 6).

**TABLE 6:** Tick‐borne haemopathogen species prevalence and seasonal dynamics analyzed with univariable logistic regression.

Type of TBHP	Season	OR	SE	Prevalence (%)	95% CI	*p*‐value
*B. bigemina*	Dry^a^					
	Short rainy	3.23	1.23	10	1.53–6.83	0.002^b^
*A. marginale*	Dry^a^					
	Short rainy	6.51	4.11	5.4	1.88–22.46	0.003^b^
*A. centrale*	Dry^a^					
	Short rainy	1.51	1.38	1.3	0.25–9.12	0.655
*Theileria *sp.	Dry^a^					
	Short rainy	5.22	4.07	3.1	1.13–24.12	0.035^b^
Overall				17.1		

Abbreviations: CI = confidence interval, OR = odds ratio, SE = standard error, TBHP  = tick‐borne hemopathogen.

^a^ reference category.

^b^ statistically significant.

#### 3.7.2 Risk Factors Associated With Tick‐Borne Haemopathogens Infection

Risk factors, including season, district, production system, BCS, breed, age, sex and tick infestation, were considered in univariable logistic regression analysis. Among these factors, season, district, breed, age, sex and tick infestation were significantly associated with tick‐borne haemopathogens (Table 7). These significantly associated factors were further analysed by multivariable logistic regression, as shown in Table 8. Consequently, season, breed, age, sex and tick infestation had statistically significant associations with the occurrence of tick‐borne haemopathogens. The short rainy season was 4.59 times more likely to risk the occurrence of tick‐borne haemopathogens than the dry season. Local breeds were 0.35 less likely at risk of tick‐borne haemopathogens than crossbreeds. Likewise, haemopathogens in terms of age, sex and tick‐infestation were 2.79, 2.28 and 8.78 times more likely to occur in adult, female and tick‐infested cattle than in young, male and non‐tick‐infested cattle, respectively (Table 8).

**TABLE 7:** Univariable logistic regression analysis of risk factors associated with tick‐borne hemopathogens.

Risk factors	Category	No. of animals examined	No. of positive animals (prevalence %)	COR (95% CI)	*p* value
Season	Dry^a^	196	14 (7.1)		
	Short rainy	196	53 (27)	4.82 (2.57–9.03)	0.000^b^
District	Bahir Dar	145	32 (22.1)		
	Zuria^a^				
	Guangua	247	35 (14.2)	0.58 (0.34–0.99)	0.046^b^
Production system	Extensive^a^	207	33 (15.9)		
	Ranch	173	31 (17.9)	1.15 (0.6–1.97)	0.608
	Semi‐intensive	12	3 (25)	1.76 (0.45–6.84)	0.416
BCS	Good^a^	30	4 (13.3)		
	Medium	271	53 (19.6)	1.58 (0.53–4.72)	0.413
	Poor	91	10 (11)	0.80 (0.23–2.78)	0.728
Breed	Cross^a^	44	15 (34.1)		
	Local	348	52 (14.9)	0.34 (0.17– 0.68)	0.002^b^
Age	Young^a^	112	10 (8.9)		
	Adult	280	57 (20.4)	2.61 (1.23–5.31)	0.008^b^
Sex	Male^a^	136	12 (8.8)		
	Female	256	55 (21.5)	2.83 (1.46–5.49)	0.002^b^
Tick infestation	Negative^a^	48	1 (2.1)		
	Positive	344	66 (19.2)	11.16 (1.51–82.35)	0.018^b^

Abbreviations: CI = confidence interval, COR = crude odds ratio.

^a^ reference category.

^b^ statistically significant.

**TABLE 8:** Multivariable logistic regression analysis of risk factors associated with tick‐borne hemopathogens.

Risk factors	Category	No. of animals examined	No. of positive animals (Prevalence %)	AOR(95% CI)	*p*‐value
Season	Dry^a^	196	14 (7.1)		
	Short rainy	196	53 (27)	4.59 (2.36–8.95)	0.000^b^
Breed	Cross^a^	44	15 (34.1)		
	Local	348	52 (14.9)	0.35 (0.14–0.89)	0.027^b^
Age	Young^a^	112	10 (8.9)		
	Adult	280	57 (20.4)	2.79 (1.29–6.04)	0.009^b^
Sex	Male^a^	136	12 (8.8)		
	Female	256	55 (21.5)	2.28 (1.12–4.65)	0.023^b^
Tick infestation	Negative^a^	48	1 (2.1)		
	Positive	344	66 (19.2)	8.78 (1.11–69.53)	0.040^b^

Abbreviations: AOR = adjusted odds ratio, CI = confidence interval.

^a^ reference category.

^b^ statistically significant.

## 4 Discussion

The impact of ticks is ranked high in Africa (Nyangiwe et al. 2018). For instance, in Ethiopia, ticks cause a financial loss to the livestock industry, accounting for 75% of animal exports (Zeleke and Bekele 2004), and approximately 53419USD losses annually through the rejection and downgrading of hides and skins (Ashenafi et al. 2013). In the current study, an overall prevalence of 87.76% tick infestation was recorded in cattle. Almost similar findings were reported by Ayana et al. (2021) in the Yabello district (89.89%), Nemomsa and Morka (2019) in Jardega Jarte District (87%) and Beyene et al. (2015) in Bedelle district (82%). However, the current finding was slightly lower than the report of Dabasa et al. (2017) in Dillo district (98.2%) and Seid and Mohammed (2017) in and around Chiro, Oromia region (96.4%). In contrast to this, the present finding is higher than the findings of Adugna and Tamrat (2022) in Ankasha and Jawi districts (45%), Fentahun et al. (2023) in Areka district, Wolaita (71.6%); Wondimu and Bayu (2021) in Haramaya Eastern Hararghe (34.3%), Lemu et al. (2023) in Bedelle district (71.9%) and Kemal and Abera (2017) in Dassenech District, Southern Ethiopia (72.1%). This variation in tick prevalence might be due to the variation of biotic (presence of suitable hosts and vegetation) and abiotic (humidity and temperature) factors of the study areas as well as variation in sampling seasons.

*Amblyomma*, *Rhipicephalus*, *Hyalomma* and *Rhipicephalus* (*Boophilus)* genera, and *Rh. (B.) decoloratus*, *A. variegatum*, *A. lepidum*, *Rh. evertsi* and *Hy. rufipes* species were detected. Our finding is in agreement with Ayana et al. (2016), Kebede et al. (2018), Lemu et al. (2023) and Huruma et al. (2015), who reported similar tick genera in North Gondar, Central Oromia, Bedelle district and Sebeta, respectively.

Out of the total 5039 ticks collected from 344 cattle, *Rh. (B.) decoloratus* was the most dominant (38%) tick species. The second and third most abundant tick species were *A. variegatum* and *Rh. evertsi*, respectively. The tick infestation burden was significantly (*p* < 0.05) different between seasons and districts. The tick count per animal was significantly higher (*p* < 0.001) during the short rainy season than during the dry season. This seasonal variation might be due to the effect of abiotic factors like relative humidity and temperature on the life cycle of ticks (Domatskiy and Sivkova 2023). Dry environmental conditions are a serious problem for tick survival, particularly for the questing larvae which are very susceptible to drying out fatally (Walker et al. 2014). In addition to this, the questing behaviours of ticks are largely regulated by environmental factors such as temperature, humidity and vegetation (Di et al. 2023).

The tick count per animal was also significantly (*p* < 0.001) higher in the Guangua district than in the Bahir Dar Zuria district. This difference might be due to variations in abiotic (humidity, temperature) and biotic (vegetation) factors which can shape the ecology of ticks, and the presence of suitable hosts (Borşan et al. 2020).

Even though ticks are not site specific most of them prefer specific body parts of animals. In the present study, each species of ticks was collected from different body parts of animals, but specific species of ticks mainly dominate at specific body parts of animals. For example, *Rh. (B.) decoloratus* were mainly found in dewlap and *A. variegatum* were mainly found in the groin/udder/scrotum. Similarly, *Rh. evertsi* and *Hy. rufipes* were primarily found under the tail and anal areas. The same evidence is also reported by (Addo et al. 2024) in Ghana. This is due to the preference of ticks on body parts of animals which have thin skin and good blood flow as suggested by (Hurtado and Giraldo‐Ríos 2018). In addition to this site preference might be an evasion mechanism from sunlight desiccation and natural enemies.

The overall male‐to‐female ratio of ticks collected revealed a higher number of males than females. The sex ratio (M:F) was greater than one in all species of ticks, except *Rh. (B.) decoloratus*. This finding is in line with the report of Mideksa et al. (2017) in Central Ethiopia, Lemu et al. (2023) in Bedelle district and Aboma et al. (2017) in Southwest Ethiopia. This might be associated with fully engorged female ticks dropping to the ground to lay eggs, whereas male ticks stay on the host for a longer time and continue feeding and mating with other female ticks (Chali et al. 2017). In contrast, the number of male *Rh. (B.) decoloratus* was lower than that of females. This finding is in agreement with the report of Lemu et al. (2023). This lower male‐to‐female ratio might be due to their small size, which makes them difficult to detect and count.

Tick‐borne haemopathogens are important proportions of all animal diseases that affect the livelihood of poor farmers in tropical countries FAO (2011), and they contribute the most among animal diseases in Ethiopia (Abunna et al. 2009). The present study revealed an overall prevalence of 17.1% tick‐borne haemopathogens. This finding is roughly comparable with previous reports of 11.4% in West Arsi, Central Ethiopia by Bariso and Worku (2018). However, there are also reports which are lower and greater than the present findings in different parts of the country. For instance, Shane et al. (2017) and Sitotaw et al. (2014) reported 2.1% in Tiyo district and 3.9% in and around Debrezeit, Central Ethiopia, respectively, from blood smears, and Hailemariam et al. (2017) reported 96.9% tick‐borne haemopathogens in Southwest Ethiopia by molecular technique. This variation might be due to variations in tick distribution and abundance as well as variations in the sensitivity of diagnostic techniques used for the examination of haemopathogens.

In the present study, *B. bigemina*(10%), *A. marginale* (5.4%), *Theileria sp*. (3.1%) and *A. centrale* (1.3%) were detected in descending order of their prevalence.

Tick‐borne haemopathogens had statistically significant differences between dry and short rainy seasons. *Babesia bigemina*, *A. marginale* and *Theileria*sp. were 3.23, 6.51 and 5.22 times more likely to occur in the short rainy season than in the dry season, respectively. These seasonal variations might be most likely associated with the abundance of vector ticks, as suggested by Kamani et al. (2010) and evidenced in the present study, whereby higher infestation of ticks was observed in the short rainy season. The maximum incidence of tick‐borne haemopathogen infection occurred soon after the peak of the tick population (Kamani et al. 2010).

Season, breed, age, sex and tick infestation were significantly associated with tick‐borne haemopathogen infections. Cattle were 4.59 times more likely to be at risk of tick‐borne haemopathogen infection in the short rainy season than in the dry season. This might be due to the increment in tick population which can play a great role in tick‐borne haemopathogen transmission during the short rainy season. Local breeds of cattle were 3.5 times less likely to be at risk of tick‐borne haemopathogens infection than crossbreeds. Similar findings were reported by Rahbari et al. (2008), who showed that local breeds are more resistant to tick‐borne haemopathogens than crossbreed cattle. This could be due to natural selection and indigenous breeds were living for a long period of time with ticks and tick‐borne haemopathogens, as a result, they have developed innate resistance to tick infestation and tick‐borne haemopathogens (Maharana et al. 2016). Adult cattle were 2.79 times more likely to be at risk of tick‐borne haemopathogens than young cattle. The possible reasons that young cattle are relatively resistant to tick‐borne haemopathogen infection is the passive transfer of maternal antibodies via colostrum as suggested by Sitotaw et al. (2014) and relatively less exposure to vector ticks as evidenced by current findings. Likewise, female cattle were 2.28 times more likely to be at risk than male cattle. The most likely reason might be female cattle's immune system is weakened due to hormonal disturbances, its use for milk production, draught power and the breeding system as suggested by Sitotaw et al. (2014). Moreover, tick‐infested cattle were 8.78 times more likely to be at risk of tick‐borne haemopathogen infection than tick‐non‐infested cattle. This is directly associated with the vectorial role of ticks for tick‐borne haemopathogen transmission.

## 5 Conclusion

In the present study, the overall prevalence of tick infestation was 87.8%, and approximately 4329 adult and 710 immature ticks of four genera and five species were collected from 344 cattle during the study period. Among these, *Rh. (B.) decoloratus* species was the most prevalent and abundant, whereas *A. lepidum* was the least prevalent and abundant. Most tick species were significantly increased in their number during a short rainy season compared with the dry season. Breed, sex, age and district had statistically significant associations with tick infestations. In addition to tick infestation, 67 cattle were also infected with one or more species of tick‐borne haemopathogens with an overall prevalence of 17.1%. *Babesia bigemina*, *A. marginale*, *A. centrale* and *Theileria* species were detected. *Babesia bigemina*, *A. marginale* and *Theileria* species were significantly associated with season, breed, age, sex and tick infestation. In general, both ticks and tick‐borne haemopathogens were increased during the short rainy season than the dry season. Therefore, further studies should be conducted for the remaining seasons and detecting tick‐borne haemopathogens with molecular techniques.

## Author Contributions

**Aschalew Shitu Yenew**: conceptualization, data curation, formal analysis, investigation, methodology, software, validation, visualization, writing – original draft, writing – review and editing. **Shimelis Dagnachew Nigatu**: conceptualization, data curation, formal analysis, funding acquisition, project administration, resources, software, supervision, visualization and writing – review and editing. **Zewdu Seyoum**: data curation, formal analysis, funding acquisition, resources, software, supervision, validation, visualization, writing – review and editing. **Mersha Chanie**: data curation, formal analysis, funding acquisition, resources, software, validation, writing – original draft, writing – review and editing.

## Ethics Statement

This work was approved by Animal research ethical review committee of University of Gondar, College of Veterinary Medicine and Animal Sciences before starting the study. The consent of animal owners was received and the ethical standard was maintained to minimise pain or discomfort for animals during the study period.

## Conflicts of Interest

The authors declare no conflicts of interest.

### Peer Review

The peer review history for this article is available at https://www.webofscience.com/api/gateway/wos/peer‐review/10.1002/vms3.70293

## Data Availability

The data that support the findings of this study are available from the corresponding author upon reasonable request.
